# Risk Factors for Neuropathic Pain in Digital Amputations

**DOI:** 10.3390/jcm15020539

**Published:** 2026-01-09

**Authors:** Alessandro Crosio, Pierpaolo Caputo, Maria Carolina Fra, Luca Monticelli, Monica Cicirello, Julien Teodori, Giulia Colzani, Alessandro Fenoglio, Davide Ciclamini, Paolo Titolo, Bruno Battiston

**Affiliations:** 1 Department of Hand Surgery and Reconstructive Microsurgery, Trauma Hospital, Città Della Salute e Della Scienza di Torino, Via Zuretti 29, 10126 Torino, Italy; 2 Department of Hand Surgery, Pellegrini Hospital, ASL NAPOLI 1, Via Portamedina alla Pignasecca, 80134 Naples, Italy; 3 Department of Hand Surgery, Ospedale Borgo Trento of Verona, AOUI Veron, Piazzale Aristide Stefani, 37126 Verona, Italy; 4 Orthopedics and Traumatology, AOU Maggiore Della Carità Hospital, Corso Mazzini 18, 28100 Novara, Italy

**Keywords:** painful neuroma, digital amputations, cold intolerance, nerve repair, neuropathic pain, peripheral nerve injury

## Abstract

**Background/Objectives:** Finger amputation is frequently followed by complications, with reported revision rates of up to 20%. One of the most disabling sequelae is the formation of painful neuromas, occurring in approximately 3–9% of cases. Several biological and mechanical risk factors have been proposed, but the potential influence of psychological traits remains poorly understood. This study aimed to investigate whether a correlation exists between patients’ personality traits and the development of neuropathic pain or related symptoms. **Methods:** A retrospective study was conducted at a Level II Hand Trauma Center, including patients who underwent digital amputation between 2021 and 2023. Neuropathic pain and cold intolerance were assessed using the S-DN4 and CISS questionnaires, respectively. Personality traits were evaluated using the BFI-10 scale. Demographic data and other clinical risk factors, including work-related injuries, psychiatric history, infection, treatment delay, and surgical technique, were also analyzed. **Results:** A total of 54 patients were included. Neuropathic pain, defined by an S-DN4 score ≥ 4, was identified in 10 patients (18.5%). A significant correlation was found between the occurrence of neuropathic pain, cold intolerance, and the “neuroticism” personality trait. Patients with work-related injuries or psychiatric disorders also showed a higher risk of neuropathic pain and cold intolerance. Conversely, infection and delayed treatment were associated with an increased risk of revision procedures, whereas the type of surgical technique used for nerve stump management was not significantly correlated with pain outcomes. **Conclusions:** The study demonstrated a meaningful association between the neurotic personality trait and both neuropathic pain and cold intolerance after finger amputation. Additionally, work-related injuries and psychiatric comorbidities were identified as potential risk factors. Patients exhibiting these characteristics may benefit from early psychological assessment and multidisciplinary management to prevent further complications and improve postoperative outcomes.

## 1. Introduction

Fingertip injuries represent one of the most common types of hand trauma, accounting for approximately 4.8 million emergency department visits each year [1]. The choice of treatment following a digital amputation depends on several factors, including the characteristics of the injury, patient-specific factors, institutional resources, the patient’s preferences, and regional or cultural considerations. The primary goal of revision amputation is to preserve digit length and aesthetic appearance while ensuring a functional and pain-free outcome [2].

Revision amputation remains a reliable option for patients who either do not meet the criteria for replantation or refuse this option [3]. Available treatments range from conservative approaches, such as semi-occlusive dressings, to more complex reconstructive procedures, including flap coverage or reimplantation. However, all these interventions carry the risk of postoperative complications, with reported revision rates of up to 20% [4].

The most frequent complications include joint stiffness, painful scarring, cold intolerance, generalized tenderness, and neuropathic pain. Cold intolerance is a common sequela following nerve injury and is characterized by thermal hyperalgesia and/or allodynia. Reported prevalence ranges from 2% to 53% after nerve repair and 4% to 8% following finger amputation. Neuropathic pain, defined as pain arising without the presence of noxious stimuli, has an estimated overall prevalence of 7–10%, with rates reaching up to 20% in patients with hand trauma. These complications may have a deep impact on daily life, delaying return to work and reducing quality of life, with considerable economic implications both for patients and healthcare systems [5,6].

Multiple factors are known to influence the development of neuropathic pain, including smoking, age, and delayed treatment [1]. Moreover, growing evidence supports a mind–body interaction, with several studies reporting an association between psychiatric disorders and neuropathic pain [7].

Neuroimaging and animal studies demonstrate that neuropathic pain induces maladaptive neuroplastic changes in brain regions involved in both sensory and affective processing, such as the anterior cingulate cortex, insula, hippocampus, and prefrontal cortex. These changes are associated with cognitive and emotional disturbances, including depression, anxiety, and impaired memory, which are highly prevalent in patients with neuropathic pain [8]. Psychological traits such as pain catastrophizing, anxiety, and personality factors (e.g., neuroticism) are shown to modulate pain sensitivity and neuroplasticity. Multimodal MRI studies reveal that individual differences in pain sensitivity are mediated by covarying structural and functional brain patterns, which are themselves influenced by psychological factors [9].

Recognizing that each patient presents unique psychological and biological characteristics, we sought to investigate whether specific personality traits might predispose individuals to these complications.

The aim of the present study was therefore to evaluate the potential association between personality traits and the development of neuropathic pain or cold intolerance following finger amputation. Additionally, we explored other clinical and demographic variables within our cohort to identify further potential risk factors.

## 2. Materials and Methods

Patients who underwent amputation surgery at the DIP, PIP, or MCP levels from 1 January 2021, to 28 February 2023, at the Hand and Upper Limb microsurgical department of our institution were included in the study.

Exclusion criteria were as follows: age under 18 years, follow-up duration of less than one year, and amputation at a level more proximal than the metacarpal bones.

For each patient demographic characteristics (age, sex, smoking status, educational level, occupation, dominant hand before and after surgery), level of amputation, type of injury, whether the trauma occurred at work, time elapsed between trauma and surgery, whether the surgery was performed in the emergency room or in the operating theater, which nerve treatment and if further procedures were done were retrieved from medical reports.

Each patient was seen for a new visit during which neuropathic pain, cold intolerance and psychological traits were assessed with S-DN4, CISS-it, and BFI scores.

The Short Douleur Neuropathique 4 (S-DN4) questionnaire was used to assess the presence of neuropathic pain with high sensitivity and specificity. The total score is 7, with a score of 0 indicating the absence of pain. According to validation studies by Van Seventer et al., a score of 4 or higher is considered indicative of neuropathic pain. Patients scoring less than 4 points on the DN4 were classified as having “non-neuropathic pain”. (2, 3) [10].

The CISS-it test was used to assess Cold intolerance. The CISS-it score ranges from 0 to 100, with a cut off ≥30/100 indicating pathological cold intolerance.

CISS scores have been arbitrarily grouped into mild (4–25), moderate (26–50), severe (51–75) and extreme severe cold intolerance (76–100) [11,12].

The BFI-10 is a 10-item scale measuring the Big Five personality traits: Extraversion, Agreeableness, Conscientiousness, Emotional Stability, and Openness. In our study, we used the Italian version of the BFI-10 proposed by Guido et al. in 2014 [13], which was originally developed in English and German by Rammstedt and John (2007) [14].

### Statistical Analysis

Assumptions of normality of variance were assessed using the Shapiro–Wilk test. Where these assumptions were met, association between continuous variables was analyzed with Spearman test, linear regression analysis and Mann–Whitney U test.

Non-continuous variables were tested by the Fisher exact test.

Associations between multiple variables was studied using the regression multivariate analysis and the ordinal logistic regression.

A *p*-value of smaller than 0.05 was considered statistically significant.

Generative artificial intelligence (GenAI) has been used in this paper to assist in data analysis.

The study was conducted in agreement with Helsinki’s declaration.

## 3. Results

Database analysis retrieved a total of 87 patients treated for fingertip amputation. Among these patients, telephonically interviewed, 54 of them agreed to participate. Patients were then divided in groups according to the S-DN4 score (above or below 4) and CISS score of (above or below 30). Characteristics of each group are summarized in Table 1.

The percentage of patients with neuropathic pain, identified through a S-DN4 score of 4 or greater, was 18.5% (10 patients). Twenty-three patients (42.6%) had a CISS score greater than 30, indicating pathological cold intolerance (summarized in Table 1).

### 3.1. Personality Traits

**Figure 1:**
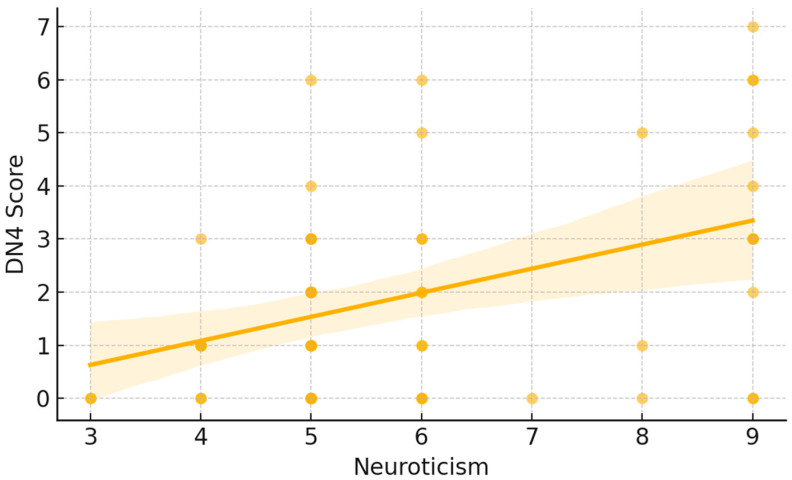
Correlation between Neuroticism and DN4 Score.

**Figure 2:**
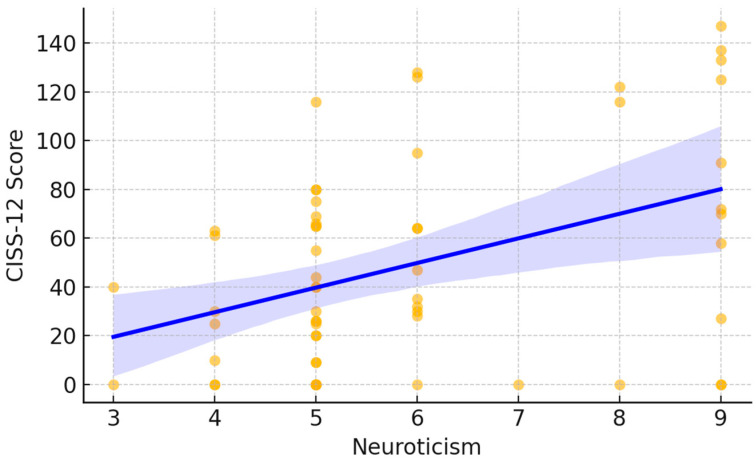
Correlation between Neuroticism and CISS-12 Score.

A moderate positive correlation emerged between neuroticism and neuropathic pain symptoms as measured by the S-DN4. Spearman’s rank correlation showed a significant direct relationship (ρ = 0.35, *p* < 0.05). As illustrated in Figure 1, each point represents an individual participant’s DN4 score plotted against their neuroticism score. The fitted trend line demonstrates an overall upward pattern, indicating that higher neuroticism scores tended to coincide with higher DN4 values. Visually, lower neuroticism scores were mostly associated with DN4 values near the lower end of the scale, whereas higher neuroticism scores showed a wider dispersion and included the highest DN4 observations. A similar pattern was observed for cold intolerance. Neuroticism was significantly and positively correlated with CISS-12 scores (Spearman ρ = 0.36, *p* < 0.05). In Figure 2, participants with higher neuroticism scores more frequently exhibited higher CISS-12 values, with the largest cold-intolerance scores occurring predominantly at the upper end of the neuroticism range. The fitted line and its shaded confidence band summarize this increasing trend while also highlighting the variability in CISS-12 scores across neuroticism levels.

### 3.2. Time

The Spearman test showed no association between delay in treatment and degree of S-DN4 and CISS whereas linear regression analysis showed a very slight correlation with minimal clinical correlation.

### 3.3. Type of Injury

**Figure 3:**
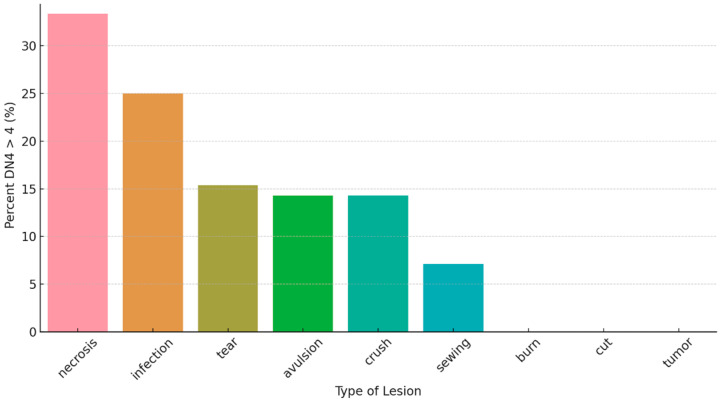
Proportion of patients with neuropathic pain (DN4 > 4) within each lesion type; bars represent within-group percentages.

Fisher’s exact test showed no significant association between Type of injury and neuropathic pain or cold intolerance. However, Figure 3 shows that DN4 > 4 was most frequent in amputations due to necrosis and infection, while other mechanisms showed lower percentages; categories with very few cases showed no DN4 > 4 events, likely reflecting small subgroup sizes.

### 3.4. Biological Characteristics of Patients

**Figure 4:**
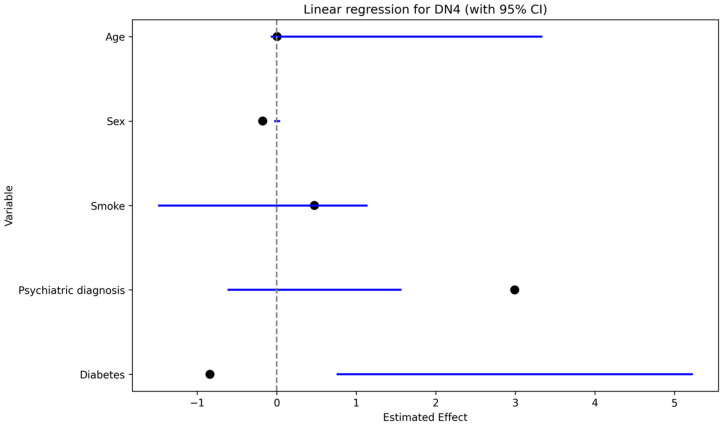
Effect of Variables on S-DN4 Score (Linear Regression, Merged Sex).

Neuropathic pain (DN4 > 4) was significantly associated with a history of psychiatric disorder in univariate analysis (Fisher’s exact test, *p* < 0.05). In the multivariable linear regression with S-DN4 score as the dependent variable (Figure 4), psychiatric diagnosis showed the largest positive coefficient estimate, followed by work-related injury and smoking; sex showed a negative coefficient, while age had a minimal effect.

### 3.5. Work

**Figure 5:**
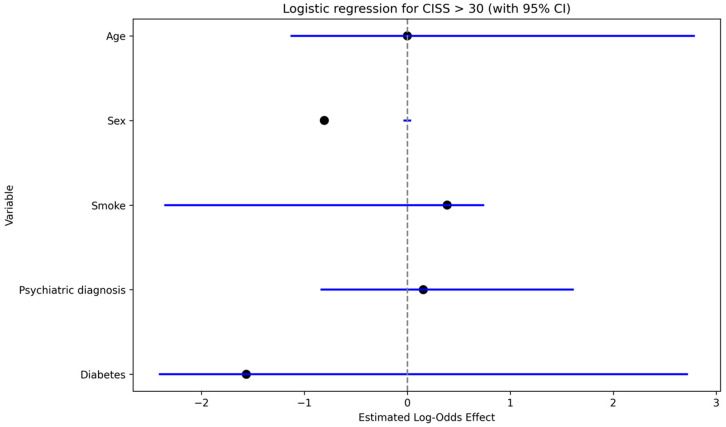
Logistic regression coefficients (log-odds) for CISS > 30 (sex merged); values > 0 indicate increased odds relative to the reference category.

Work-related injuries were significantly associated with neuropathic pain (DN4 > 4) on univariate analysis (Fisher’s exact test, *p* < 0.05). In multivariable models, work-related injury showed a positive effect estimate on S-DN4 score (linear regression; Figure 4) and was associated with increased odds of clinically relevant cold intolerance (CISS > 30) (logistic regression: OR 3.7, *p* < 0.05; Figure 5).

### 3.6. Type of Surgical Procedure

**Figure 6:**
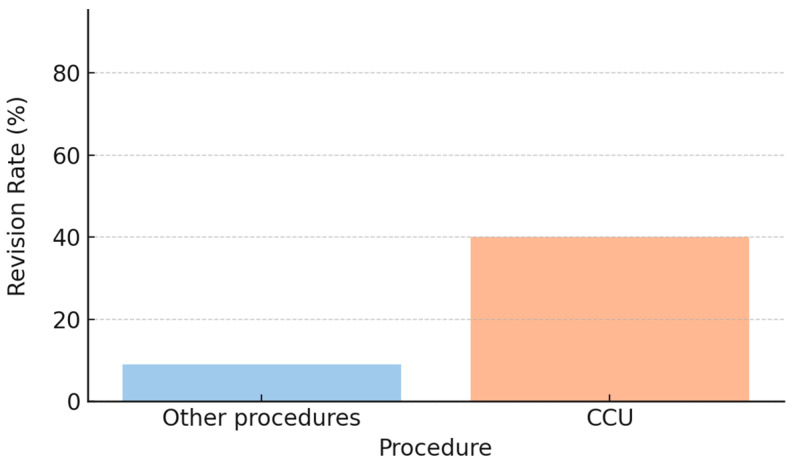
Revision Rate: CCU vs. Other Procedures.

**Figure 7:**
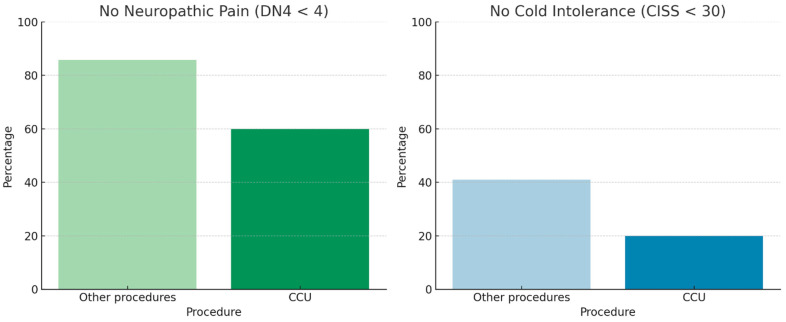
Neuropathic Pain and Cold Intolerance correlation with CCU or Other procedures.

Neuropathic pain and cold intolerance were not associated with whether the amputation was performed in the emergency department or in the operating room. Nerve management techniques included cauterization, transection, or termino–terminal neurorrhaphy of the two collateral nerves (CCU). In our cohort, nerve transection was associated with lower rates of neuropathic pain and cold intolerance (Fisher’s exact test, *p* < 0.05). In contrast, patients treated with CCU showed higher revision rates (approximately 40% vs. 10% for the other procedures; Figure 6), along with poorer descriptive outcomes, characterized by a lower proportion of patients free from neuropathic pain (DN4 < 4) and cold intolerance (CISS < 30) (Figure 7). However, the association between CCU and revision surgery did not reach statistical significance (Fisher’s exact test, *p* = 0.09).

### 3.7. Risk of Revision

In our cohort of patients, revision was defined as another surgical procedure required due to neuropathic pain after initial terminalization.

Regression multivariate analysis was performed to investigate the association between revision surgery and type of injury, time between injury and first surgical procedure and type of procedure performed on the nerve.

**Figure 8:**
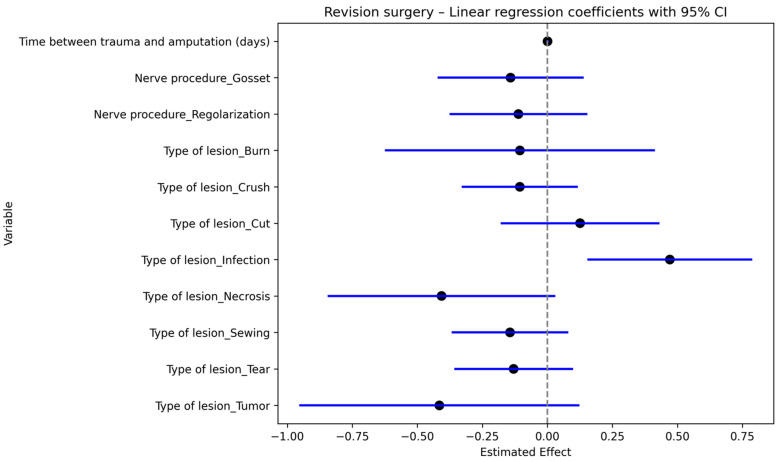
Regression Coefficients for Revision Procedure.

Delay in first treatment and infection showed higher rates of revision surgery (*p* = 0.00 and *p* = 0.04, respectively) Figure 8. Meaning that the more the time passed between trauma and amputation, the higher was the risk of revision.

Therefore, a significant association between revision and presence of neuropathic pain was also shown by Fisher’s test (*p* < 0.05).

### 3.8. Ordinal Logistic Regression Analyses

Ordinal logistic regression models (proportional odds) were used to examine clinical, demographic, and psychological predictors of neuropathic pain severity (S-DN4) and cold intolerance severity (CISS-it). Across both models, neuroticism emerged as the only independent and consistent predictor of symptom severity. Specifically, higher neuroticism scores were associated with increased odds of belonging to higher severity categories for neuropathic pain (OR = 1.47, *p* = 0.012) and cold intolerance (OR = 1.53, 95% CI 1.12–2.13, *p* = 0.009). For neuropathic pain severity, the interval between trauma and amputation demonstrated a non-significant trend toward higher S-DN4 categories (*p* = 0.099), suggesting a possible association between delayed surgical intervention and greater neuropathic pain. Although psychiatric diagnosis, work-related injury, and the presence of comorbidities did not reach statistical significance, all non-significant predictors showed positive coefficients in the multivariable model, indicating a consistent direction toward increased neuropathic pain severity. These findings suggest potential contributions of these factors that may not have been detectable due to limited statistical power or residual confounding. In contrast, none of the examined clinical or demographic variables, aside from neuroticism, independently predicted cold intolerance severity. The estimated effect of time between trauma and amputation was minimal and close to null. Work-related injury and comorbidities showed non-significant odds ratios greater than one, but these estimates were accompanied by wide confidence intervals, reflecting substantial uncertainty. Psychiatric diagnosis was not associated with cold intolerance severity and yielded a particularly imprecise estimate, likely due to the small number of individuals with this characteristic.

Overall, these analyses indicate that neuroticism is a robust independent predictor of both neuropathic pain and cold intolerance severity, whereas other clinical and demographic factors did not demonstrate statistically significant independent effects in this sample. Nonetheless, the observed directional trends, particularly for neuropathic pain, underscore the need for larger, prospective studies to better clarify the potential role of treatment delay and other contextual factors.

## 4. Discussion

Van der Avoort et al. reported a post-traumatic neuropathic pain incidence of 7.8% in patients with finger amputations, while other studies have described rates of painful neuroma formation after upper-extremity amputation ranging from 4% to 25% [15,16]. In our cohort, which included only digital amputations, similar rates were observed, confirming the comparability of our population with previously reported data.

Given the high incidence of this complication, numerous preventive strategies have been proposed; however, no single technique has yet been universally accepted as the most effective [5].

Cold intolerance, reported in 4–8% of digital amputations [6], is a disabling sequela that can significantly limit hand function and may coexist with neuropathic pain. It also represents one of the most frequent complaints after digital replantation [17].

To our knowledge, this is the first study investigating the relationship between personality traits and neuropathic pain following digital amputation. In our cohort, higher levels of neuroticism were linearly associated with both neuropathic pain and cold intolerance, indicating that individuals with elevated neuroticism may be more prone to pain perception. It remains unclear, however, whether neuroticism predisposes individuals to neuropathic pain or whether pain itself modifies personality traits. The literature supports the possibility that personality can change following traumatic events [18,19]. Prospective longitudinal studies are needed to clarify this relationship. Our institution is planning a prospective study in which personality traits will be assessed at admission and re-evaluated during follow-up.

These findings reinforce the concept of a mind–body interaction in painful neuromas, as previously suggested by Hainline [7].

Our results also support a close link between cold intolerance and neuropathic pain, extending previous findings by Magistroni et al. [12]. The association between neuropathic pain and psychiatric conditions further emphasizes this connection. Notably, in our series, patients with psychiatric diagnoses often exhibited neuropathic pain that predated the traumatic event. It is therefore plausible that pre-existing mental health disorders may predispose individuals to symptomatic neuroma development, consistent with the observations of Vieira et al. [20].

An additional finding of interest was the significant association between work-related injuries and both neuropathic pain and cold intolerance. The underlying mechanism remains unclear. Although similar associations between occupational factors and neuropathic pain have been reported [21], a direct link between work-related injuries and painful neuroma formation has not been previously demonstrated. In our cohort, most occupational injuries were treated promptly; however, some represented complications of previous interventions, which may confound interpretation. Psychological stress related to occupational trauma could contribute to this association, though further targeted research is required.

Regarding surgical factors, no differences in neuropathic pain or cold intolerance were observed between procedures performed in the operating room and those performed in the emergency department, suggesting that the setting of initial revision amputation does not influence sensory outcomes. This contrasts with the findings of Gil et al. [6], who demonstrated cost-effectiveness advantages for emergency department procedures.

Consistent with previous reports, injury type significantly influenced the incidence of neuropathic pain. Our cases included lacerated–contused wounds, avulsions, crush injuries, and infections. The discrepancy observed between optimal and uncertain surgical outcomes in guillotine and avulsion injuries aligns with the literature, which links avulsion amputations with higher neuroma risk [6,16]. Our findings further suggest that infection and necrosis favor neuroma formation, supporting the hypothesis that local inflammation sustains and promotes painful neuroma development, as described in both clinical and preclinical studies [22,23].

Although techniques such as neurorrhaphy, flap coverage, and epineural grafting have shown variable success, no method has emerged as a definitive standard. Some studies have reported that coaptation of digital nerve ends may reduce neuroma formation [2], while others found conflicting results. In our series, the use of certain techniques, such as CCU, was associated with a higher revision rate, likely reflecting delayed treatment or infection rather than procedural failure.

Lange et al. [1] found that, in patients undergoing digital nerve repair, the time interval between trauma and surgery significantly influenced neuropathic pain outcomes. Specifically, a delay longer than three weeks was associated with a higher incidence of neuropathic pain, suggesting the importance of early surgical intervention [5]. Despite in our research no specific association was found between delayed treatment and neuropathic pain, delayed management and infection were associated with increased revision rates, underscoring the importance of early, appropriate intervention. This observation aligns with previous reports [1,24,25] emphasizing the need for timely surgical treatment of peripheral nerve injuries to reduce failure rates of reconstructive procedures such as targeted muscle reinnervation (TMR).

This study has several limitations that should be addressed in future prospective investigations. Neuropathic pain may be secondary to the presence of a painful neuroma; however, clear clinical signs and standardized diagnostic criteria for neuroma were not consistently available in our patient population. Therefore, our findings should be interpreted as primarily related to neuropathic pain rather than to painful neuromas per se.

Although an association with psychiatric disorders was observed, detailed information regarding the type and severity of these conditions was not available in the medical records and should be systematically collected in future studies. Moreover, as noted above, personality traits may be influenced by traumatic events. In this retrospective study, results may have been affected by such neuroplastic changes, as interviews were conducted long after the initial trauma. This potential bias could be minimized in prospective studies by administering psychological assessments at the time of admission to the emergency department.

From a methodological standpoint, no correction for multiple comparisons (e.g., Bonferroni correction) was applied, and this should be considered when interpreting the results.

## 5. Conclusions

The factors predisposing to painful neuroma formation after digital amputation remain incompletely defined. Our findings indicate that high neuroticism scores, work-related injuries, and psychiatric disorders are significantly associated with the development of painful neuromas. These patient subgroups may benefit from early psychological assessment and targeted prophylactic surgical strategies. Furthermore, infection and delayed intervention increase the risk of revision, highlighting the importance of timely and multidisciplinary management to optimize outcomes.

## Figures and Tables

**Table 1:** Patients included from database. * One patient had both injured hands, but pain only in one.

	With Painful Neuromas	Without Painful Neuromas
54 pt	10 * (18.5%)	43 (79.6%)
Mean age	45.9	49.3
Male/Female	7/2	44/8
At work	8	18
Right/Left	7/2	26/26
OR/ED	8/1	43/9
Smoker	5 (55.5%)	13 (25%)
Mean S-DN4	5.5	1.4
Mean CISS-it	71	21.5

## Data Availability

The original contributions presented in this study are included in the article. Further inquiries can be directed to the corresponding author.

## Abbreviations

The following abbreviations are used in this manuscript:

DIP	Distal interphalangeal (joint)
PIP	Proximal interphalangeal (joint)
MCP	Metacarpo–phalangeal (joint)
CCU	Centro-central union
CI	Cold intolerance
NP	Neuropathic pain
GenAI	Generative artificial intelligence
